# Risk of Bacterial Meningitis in Young Children with a First Seizure in the Context of Fever: A Systematic Review and Meta-Analysis

**DOI:** 10.1371/journal.pone.0055270

**Published:** 2013-01-28

**Authors:** Abolfazl Najaf-Zadeh, François Dubos, Valérie Hue, Isabelle Pruvost, Ania Bennour, Alain Martinot

**Affiliations:** 1 Univ Lille Nord-de-France, UDSL, Lille, France; 2 Paediatric Emergency and Infectious Diseases Unit, CHRU, Lille, France; 3 EA2694, Public Health, Epidemiology and Quality of Care, Lille, France; Washington University, United States of America

## Abstract

**Background:**

Of major concern in any febrile child presenting with a seizure is the possibility of bacterial meningitis (BM). We did a systematic review to estimate the risk of BM among various subgroups of young children with a first seizure in the context of fever, and to assess the utility of routine lumbar puncture (LP) in children with an apparent first FS.

**Methods/Principal Findings:**

MEDLINE, INIST, and the COCHRANE Library databases were searched from inception to December 2011 for published studies, supplemented by manual searches of bibliographies of potentially relevant articles and review articles. Studies reporting the prevalence of BM in young children presenting to emergency care with a first: i) “seizure and fever”, ii) apparent simple FS, and iii) apparent complex FS were included. Fourteen studies met the inclusion criteria. In children with a first “seizure and fever”, the pooled prevalence of BM was 2.6% (95% CI 0.9–5.1); the diagnosis of BM might be suspected from clinical examination in 95% of children >6 months. In children with an apparent simple FS, the average prevalence of BM was 0.2% (range 0 to 1%). The pooled prevalence of BM among children with an apparent complex FS was 0.6% (95% CI 0.2–1.4). The utility of routine LP for diagnosis of CNS infections requiring immediate treatment in children with an apparent first FS was low: the number of patients needed to test to identify one case of such infections was 1109 in children with an apparent first simple FS, and 180 in those with an apparent first complex FS.

**Conclusion:**

The values provided from this study provide a basis for an evidence-based approach to the management of different subgroups of children presenting to emergency care with a first seizure in the context of fever.

## Introduction

Seizures occur in the context of many childhood illnesses, and account for approximately 1–5% of all emergency departments (ED) visits [1], [2]. Febrile seizures (FS) represent the most common form of childhood seizures, affecting approximately 2–5% of infants and young children in Europe and North America, and 8% in Japan [3]. It is broadly defined as a seizure accompanied by fever without evidence of central nervous system (CNS) infections, occurring in children 6 months to 5 years of age [4].

The association between seizure and bacterial meningitis (BM) is well established [5]–[7]. It is therefore imperative to rule out BM prior to making the diagnosis of FS. However, the diagnosis of FS in certain subgroups of children with an apparent FS is a challenge: FS may be the sole manifestation of BM in infants [4]; complex features of seizure can increase the risk of BM in others [8], [9]. Accordingly, when these children present with an apparent FS, clinicians may remain uncertain about the risk of BM. In acute situation, the most challenging issue is to make a decision whether a lumbar puncture (LP) is necessary to rule out BM. Knowledge of the prevalence of BM among various subgroups of children with FS can assist clinicians to make appropriate clinical decisions in such challenging situations.

Numerous clinical studies reporting the prevalence of BM among children with an apparent FS have been conducted throughout the world [10]–[18]. There have been also four literature review articles (published in year 1980, 2001, 2003, and 2011) looking at the prevalence of BM in children with an apparent FS from developed countries [19]–[22]. However, the three first reviews [19]–[21] were subject to bias because of combining different patient groups in their review (i.e., children with a “seizure and fever” [8], [9], [23]–[25], those with an apparent FS [11]–[13], [26]–[31], and those with an FS [32]). The 2011 review was limited to a subgroup of children <18 months of age with an apparent first simple FS who were enrolled during post-vaccine era (i.e., immunization against *Haemophilus influenzae* type b (Hib) and *Streptococcus pneumoniae* [22].

Consequently, by using a more rigorous methodology than the previous studies, and in light of the more recent publications, we undertook a systematic review and meta-analysis, where appropriate, of the relevant literature to provide more accurate estimates of BM prevalence in young children presenting with seizure in the context of fever. The main question informing this review was: what is the prevalence of BM in young children presenting with: (i) a first “seizure and fever”, (ii) an apparent first simple FS, and (iii) an apparent first complex FS? We also sought to evaluate the utility of routine LP for diagnosis of CNS infections requiring immediate treatment among children with an apparent first FS.

## Methods

We conducted and reported this systematic review in accordance with the PRISMA (Preferred Reporting Items for Systematic reviews and Meta-Analyses) statement (Text S1) [33].

### Search strategy

The literature search aimed to identify all studies looking at the prevalence of BM in young children presenting to emergency care with a first seizure in the context of fever. To identify eligible original articles, we searched the following electronic databases from inception to December 2011: MEDLINE via PUBMED, INIST (Scientific and Technical Information Institute) via article@inist, and COCHRANE library. In each electronic database, various combinations of the following search terms were used: “febrile seizure”, “febrile convulsion”, “fever”, “seizure”, “convulsion”, “meningitis”, and “central nervous system infections”. The reference lists of potentially relevant articles and review articles were also screened for additional articles of interest. The detailed search strategy for each electronic database can be found in Text S2.

### Eligibility criteria

Explicit a priori inclusion and exclusion criteria were applied. Cohort studies on consecutive, unselected children published before 30 December 2011 were eligible for inclusion in our review if: (1) they reported data on the prevalence of BM in young children who were admitted to the ED or inpatient ward for evaluation of a first “seizure and fever”, an apparent first simple FS, or an apparent first complex FS, (2) their definition of FS (simple or complex) was the same as or very similar to that used most commonly in literature [4], [34], (3) they were performed in high-resource countries (studies from low-resource countries were excluded because of the higher prevalence of BM and the different range of conditions (i.e., malaria, HIV related CNS infections, and CNS tuberculosis) that may present as seizure with fever in such countries [35]–[39]); the United Nations list was used to define high-resource countries, and (4) they were written in English or French. When multiple articles reported on a same study population, we included only the most detailed publication that met the inclusion criteria. When studies were identified as containing pertinent data not included in the published article (e.g., studies that included children with a first FS, but did not differentiate between simple FS and complex FS) we contacted the authors to obtain missing data. When a response was not provided and raw data from the original study allowed us to differentiate between two groups of patients, such articles were included in the study; otherwise, they were excluded. Because of the likelihood that small studies would have overestimated event outcome rates, studies including less than 20 patients were arbitrary excluded. Case reports, review articles, editorials, comments, and clinical guidelines were excluded.

### Study selection process

Study selection was carried out independently by two of the reviewers (AN, AM) in two rounds. First, all titles and abstracts of the identified citations were screened. Second, potentially relevant articles were reviewed in their entirety. Each investigator made a recommendation for inclusion or exclusion of single articles. Any disagreements were resolved by consensus.

### Outcome measures and definitions

The main outcome measure was the prevalence of BM among children with a first seizure in the context of fever. Other outcome measures were: (1) the overall prevalence of CNS infections (including meningitis (viral and bacterial), “possible BM”, Herpes simplex virus (HSV) encephalitis, and encephalitis of other aetiologies) among children with a first seizure in the context of fever, and (2) the utility of routine LP for diagnosis of CNS infections requiring immediate treatment in children with an apparent first FS. CNS infections requiring immediate treatment included BM and HSV encephalitis. BM was defined by positive cerebrospinal fluid (CSF) culture results for a relevant bacterial pathogen, positive Gram's staining of CSF with a negative CSF culture, CSF pleocytosis with positive blood culture of a relevant bacterial pathogen, or CSF pleocytosis with positive latex agglutination test of CSF. “Possible BM” was defined as CSF pleocytosis with negative Gram's staining and negative results of bacterial cultures of blood and CSF in a child pre-treated with antibiotics. Pleocytosis was defined by ≥5 white blood cells per µL [22]. HSV encephalitis was defined by a positive CSF HSV polymerase chain reaction (PCR). The definition of FS was introduced in the introduction section (see above). “Seizure and fever” included any seizure in a child with fever of any cause [40]. Apparent FS was defined as an event that fulfilled the criteria used to designate an FS, but for whom the possibility of CNS infections were not yet ruled out by LP or follow-up. Simple FS was defined as a primary generalized seizure lasting less than 15 minutes and not recurring within 24 hours [4]. Complex FS was defined on one or more of the following features: a partial (focal) onset or showing focal features during the seizure, prolonged duration (greater than 10–15 minutes), and recurrence within 24 hours or within same febrile illness [34]. For each study, the period preceding routine Hib and *S. pneumoniae* vaccine implementation was considered as the pre-vaccine era and the subsequent period as the post-vaccine era [17], [41]–[43].

### Data extraction and quality assessment

Data extraction and quality assessment of the included studies were performed independently by two of the reviewers (AN, AM) using a standardized data collection form. Any disagreement was resolved unanimously by discussion. The following information was extracted from each study: first author, country of origin, dates of enrollment, type (prospective versus retrospective), clinical setting, number of patients, inclusion and exclusion criteria, method of outcome ascertainment (clinical grounds, clinical grounds with follow-up, LP), number of outcome events (CNS infections, BM), definition of FS (for studies on simple FS, and complex FS), number and outcome of patients pre-treated with antibiotics, and enrollment era (pre-vaccine versus post-vaccine era). We adapted a quality assessment system for prevalence articles [44]. Each article was reviewed to determine whether: (1) study design was appropriate for obtaining prevalence estimates, (2) sample was representative of the population of interest on key characteristics (age, medical condition), (3) a clear and acceptable definition of the outcome of interest was provided, and (4) the outcome ascertainment methods were well defined and adequate. We assessed these quality indicators separately for each article; a total quality score was not calculated [45].

### Data synthesis and analysis

All analyses were performed separately for each group of patients: children with a “seizure and fever”, those with an apparent first simple FS, and those with an apparent first complex FS. Studies, patients, outcome ascertainment methods, and outcome data were summarized using basic descriptive statistics (simple counts and proportions). The average prevalences of CNS infections and BM were calculated by dividing the number of children with target outcome by the number of children included in the study. As the prevalence of BM was the main focus of our study, the pooled proportion of BM was synthesized by using meta-analytic techniques. Proportions were first transformed into a quantity based upon the Freeman-Tukey variant of the arcsine square root transformed proportion [46] suitable for the usual fixed and random effects summaries [47]. The pooled proportion was calculated as the back-transform of the weighted mean of the transformed proportions, using inverse arcsine variance weights for the fixed effects model and DerSimonian-Laird weights for the random effects model. Statistical heterogeneity across studies was measured using the Cochran chi-square test (*p*<0.1 considered significant) and assessed visually using the Galbraith plot of heterogeneity. To determine the percentage of heterogeneity across studies, the I-squared (I^2^) statistic was calculated [48]. To evaluate the weight of particular articles on the pooled estimates, we performed influence analysis. This method recalculates the pooled prevalence estimate omitting one study at a time. Meta-regression analysis and publication bias assessment were not performed because of the small number of studies. The utility of routine LP for diagnosis of CNS infections requiring immediate treatment was estimated as “number needed to test” (NNT), denoting the number of patients who need to undergo an LP to detect one case of such infections. NNT was calculated by dividing the number of patients included in the study by the number of patients with CNS infections requiring immediate treatment who were diagnosed after routine LP. All statistical tests were performed using STATA version 11.1 (Stata Corp, College Station, Texas) and StatsDirect version 2.7.9 (StatsDirect, Ltd, UK).

## Results

### Search results

**Figure 1:**
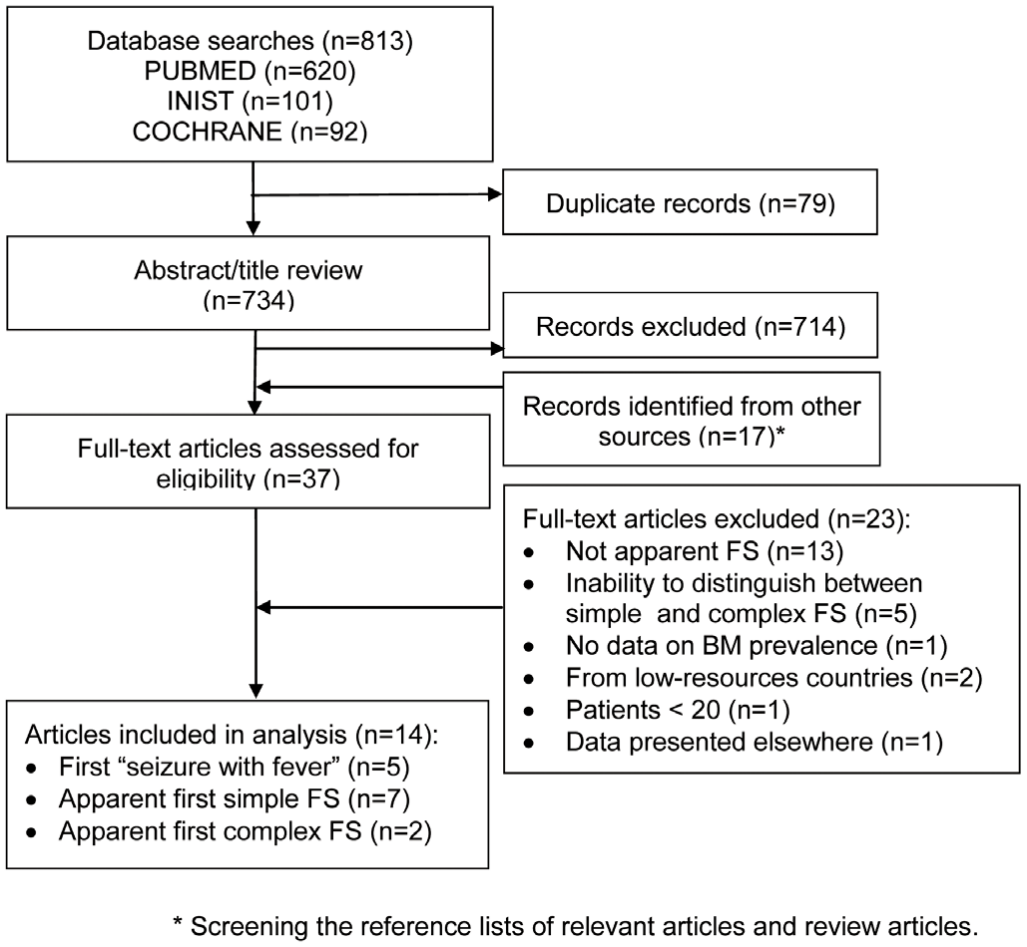
Flow diagram outlining the study selection process.

Seven hundred thirty four articles were identified through electronic database search, of which 20 were deemed relevant for full text review. Seventeen additional relevant articles were identified by screening the reference lists of relevant articles and review articles. Of these 37 articles, 14 met all criteria for inclusion. The flow of articles through the literature search and screening process, and the reasons for exclusion of the identified studies are illustrated in Figure 1.

### Children with “seizure and fever”

**Figure 2:**
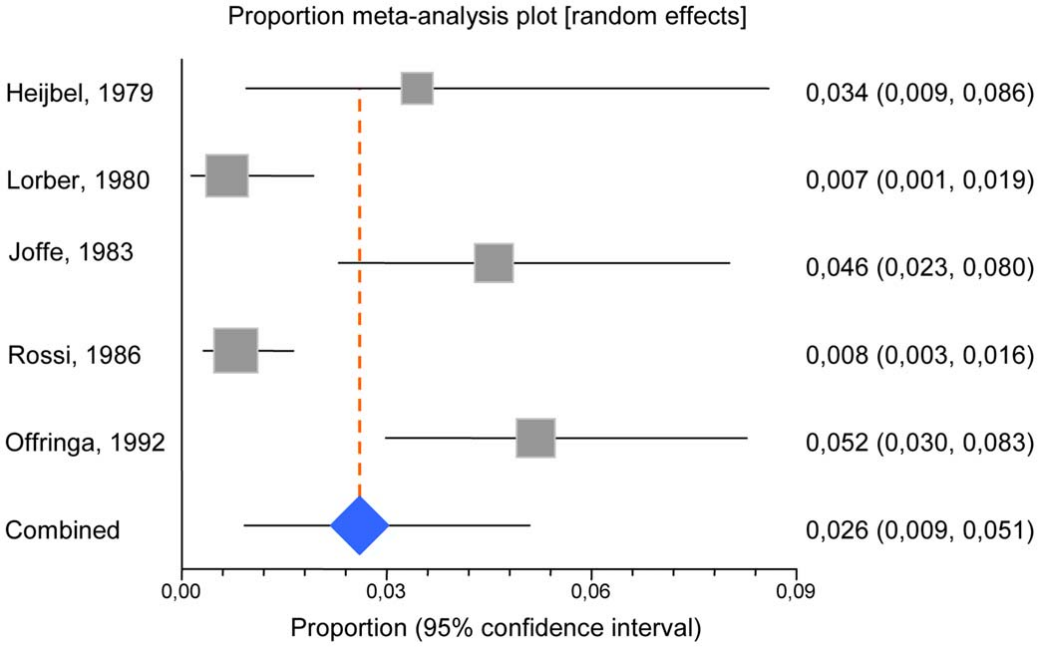
Forest plot displaying the pooled prevalence of bacterial meningitis among young children presenting with a first “seizure and fever”.

Outcome data for children with a “seizure and fever” were derived from 5 studies evaluating 1996 patients [8], [9], [23]–[25]. Characteristics, results, and methodological details of the studies are summarized in Table 1 and Table 2. In all studies, samples were representative of the population of interest on key characteristics (Table 2). Outcomes were well documented by LP or clinical examination with follow-up in 100% of patients in all studies (Table 2). Out of 1996 children, 77 were diagnosed with CNS infections, of whom 41 with BM. Of the 41 children with BM, 4 were <6 months and 37>6 months of age. The diagnosis of BM might be suspected from clinical examination in 95% (n = 35) of children >6 months (Table 1). The overall average prevalence of CNS infections was 3.9% (range 2.3 to 7.4%). The pooled prevalence of BM using a random effects model was 2.6% (95% CI 0.9–5.1) (Figure 2). When the individual studies were combined in a meta-analysis, there was significant heterogeneity among the estimates for the prevalence of BM from the studies (I^2^ = 87%, *p*<0.001). The Galbraith plot identified the populations studied by Offringa et al. and Joffe et al. as the sources of this heterogeneity [8], [9]; however, exclusion of one or both of these studies did not significantly alter the results (*p* = 0.4). Furthermore, influence analysis showed that no study, including these two, significantly impacted the pooled prevalence estimate.

**Table 1:** Characteristics and results of the studies reporting the prevalence of BM in young children with a first seizure in the context of fever.

Author/Country	Enrollment date, y	Setting	Patients, n	Age, mo	CNS infection, n			Other reported data
					BM	HSV encephalitis	Overall	
**First “seizure and fever”**								
Heijbel [23], Sweden	1973–1974	Inpatient	116a, b	6 to 60^c^	4	0	5	100% of BM patients had clinical symptoms of BM
Lorber [24], UK	1972–1976	Inpatient	452^a^	6 to 72^c^	3	0	15	100% of BM patients had either definite meningeal signs or were critically ill
Joffe [8], USA	1978–1980	2 EDs	241^a^	23^d^	11	0	13	92% of BM patients had abnormal neurological findings
Rossi [25], Italy	Before 1986	ED	878^a^	1 to 72^c^	7	0	21	100% of BM patients >6 months had significant neurological signs
Offringa [9], Netherlands	1985–1987	2 EDs	309^a^	18^e^	16	0	23	92% of BM patients had either “major” or “minor” signs of BM; possible BM: 4 cases
**First apparent simple FS**								
Jaffe [10] f, Israel	1969–1972	Inpatient	323^a^	6 to 72^c^	0	0	2	-
Gerber [11], UK	1974–1979	Inpatient	100^a^	20^d^	0	0	0	-
McIntyre [12] f, Australia	1984	Inpatient	198^a^	6 to 60^c^	2^g^	0	NS	-
Kinsella [13] f, USA	1991–1992	2 Inpatients	33^a^	16^h^	0	0	0	-
Trainor [14], USA	1995–1997	7 EDs	455^a^	21^d^	0	0	0	Children <12 months: 13% (n = 59); possible BM: 0 case
Kimia [15], USA	1995–2006	ED	704^i^	14^e^	0	0	10	Children <12 months: 27% (n = 188), of whom 109 were enrolled during post-vaccine era; possible BM: 1 case
Shaked [16], USA	2001–2005	ED	56^j^	6 to 12^c^	0	0	0	Possible BM: 0 case
**First apparent complex FS**								
Seltz [17] k, Canada	2002–2006	ED, inpatient	192 episodes^j^	19^e^	1	0	1	-
Kimia [18], USA	1995–2008	ED	526^i^	17^e^	3	0	15	Possible BM: 3 cases

BM, bacterial meningitis; CNS, central nervous system; HSV, herpes simplex virus; FS, febrile seizure; ED, emergency department; NS, not specified.

a Children were enrolled during pre-vaccine era.

b One patient was under six months of age.

c Range.

d Mean.

e Median.

f Only children with an apparent first simple FS were included in the analysis.

g Of the 2 BM cases (7 and 13 months old) 1 had a normal CSF after routine lumbar puncture (LP) but a repeated LP 24 hours later showed BM.

h The number represent the median age of the patients (n = 47) with an apparent simple FS included in the study.

i Children were enrolled during both pre and post-vaccine eras.

j Children were enrolled during post-vaccine era.

k Only consecutive, unselected children were included in the analysis.

**Table 2:** Quality indicators of the studies reporting the prevalence of BM in young children with a first seizure in the context of fever.

Author	Design	Participants	BM definition	Outcome ascertainment (%)
**First “Seizure and fever”**				
Heijbel [23]	PC, CONS	6 to 60 months with a first seizure in the context of fever	NS	LP (44), FU (56)
Lorber [24]	RC, CONS	6 to 72 months with a first seizure in the context of fever, without known neurologic disease	NS	LP (67), FU (33)
Joffe [8]	RC, CONS	6 to 72 months with a first seizure in the context of fever, without known neurologic disease	NS	LP or FU (100)
Rossi [25]	RC, CONS	1 to 72 months with a first seizure in the context of fever, without known neurologic disease	NS	LP (29), FU (71)
Offringa [9]	RC, CONS	3 to 72 months with a first seizure in the context of fever	Positive CSF bacterial culture or CSF pleocytosis ≥ 10 white blood cells per µL	LP (65), FU (35)
**First apparent simple FS**				
Jaffe [10]	RC, CONS	6 to 72 months with a first simple FS (single generalized seizure lasting <15 min, without clinical evidence of CNS infection)	NS	LP (100)
Gerber [11]	RC, CONS	6 to 60 months with a first simple FS (single generalized seizure lasting <15 min, without sign of acute neurologic disease)	NS	LP (81), FU (19)
McIntyre [12]	PC, CONS	6 to 60 months with a first simple FS (single generalized seizure lasting <15 min)	Positive CSF bacterial culture	LP or FU (90), CX (10)
Kinsella [13]	PC, CONS	2 to 61 months with a first simple FS (single generalized seizure lasting <15 min, without sign of neurologic disease)	NS	FU (100)
Trainor [14]	RC, CONS	6 to 60 months with a first simple FS (single generalized seizure lasting <20 min, T≥38°), without history of seizures or known neurologic disease	Positive CSF bacterial culture	LP (30), CX (70)
Kimia [15]	RC, CONS	6 to 18 months with a first simple FS (single generalized seizure lasting <15 min, without evidence of CNS infection), without history of seizure or trauma	Positive CSF bacterial culture, positive Gram's staining of CSF, or CSF pleocytosis with a positive blood culture	LP (38), CX (62)
Shaked [16]	RC, CONS	6 to 12 months with a first simple FS (single generalized seizure lasting <15 min, T≥38°), without history of seizure or known neurologic disease	Positive CSF bacterial culture, positive Gram's staining of CSF, or CSF pleocytosis ≥ 10 white blood cells per µL	LP (50), CX (50)
**First apparent complex FS**				
Seltz [17]	RC, CONS	6 to 72 months with a first complex FS (seizure ≥15 min, focal, or recurring within 24 h), without history of seizure, known neurologic disease, immunodeficiency, or trauma	Positive CSF bacterial culture, CSF pleocytosis with positive CSF latex agglutination test, or CSF pleocytosis with a positive blood culture	LP (45), FU or CX (55)
Kimia [18]	RC, CONS	6 to 60 months with a first complex FS (seizure ≥15 min, focal, or recurring within 24 h), without history of seizure, known neurologic disease, immunodeficiency, or trauma	Positive CSF bacterial culture or CSF pleocytosis with a positive blood culture	LP (64), FU (31), CX (5)

BM, bacterial meningitis; PC, prospective cohort; CONS, consecutive; RC, retrospective cohort; LP, lumbar puncture; FU, follow-up; CX, clinical examination; CSF, cerebrospinal fluid; FS, febrile seizure; CNS, central nervous system; NS, not specified.

### Children with an apparent simple FS

Seven studies with an aggregate of 1869 patients reported outcome data for children with a first simple FS [10]–[16]. Characteristics, results, and methodological details of the studies are summarized in Table 1 and Table 2. In all studies, samples were representative of the population of interest on key characteristics (Table 2). Outcomes were well documented by LP or clinical examination with follow-up in 100% of patients in 3 studies [10], [11], [13]. A formal meta-analysis was not done because of the excess number of zero events in the sample. The overall average prevalence of CNS infections in children 6 to 72 months of age (4 studies, n = 911) was 0.2% (range 0.0 to 1.4%), and that of BM (5 studies, n = 1109) was 0.2% (range 0.0 to 1.0%). The NNT to detect one case of CNS infection requiring immediate treatment in children 6 to 72 months of age was 1109 (of the two BM cases, one was diagnosed after routine LP and the other had a normal CSF after routine LP, but a repeated LP 24 hours later showed BM) (Table 1).

### Children with an apparent complex FS

Outcome data for children with a first complex FS were derived from two studies with an aggregate of 718 patients [17], [18]. Characteristics, results and methodological details of the studies are summarized in Table 1 and Table 2. In all studies, samples were representative of the population of interest on key characteristics (Table 2). The overall average prevalence of CNS infections was 2.2% (range 0.5 to 2.9%). The pooled prevalence of BM using a fixed effects model was 0.6% (95% CI 0.2–1.5). When the individual studies were combined in a meta-analysis, there was no significant heterogeneity among the estimates for the prevalence of BM from the studies (I^2^ = 0.0%, *p* = 0.9). The NNT to detect one case of CNS infection requiring immediate treatment was 180 (Table 1).

## Discussion

Our study is the first systematic review and meta-analysis that attempts to quantify the risk of BM in different subgroups of children with seizure in the context of fever, and to evaluate the utility of routine LP in children with an apparent first FS. The overall risk of BM was low, ranging from 0.2% in children with an apparent first simple FS to 2.6% in those with a first “seizure and fever”. The utility of routine LP for diagnosis of CNS infections requiring immediate treatment among children with an apparent first FS was low: the NNT was 1109 in children with an apparent first simple FS, and 180 in those with an apparent first complex FS.

BM may present as a seizure associated with fever [5]–[7]. In our study, 2.6% of children with “seizure and fever” were found to have BM; the diagnosis of BM might be suspected from clinical examination in 95% of children (Table 1). This figure illustrates the ability of clinical examination to identify almost all children with a first “seizure and fever” who are most likely to benefit from LP, thereby avoiding unnecessary routine LP. However, given the retrospective nature of the majority of studies, this finding will need further clinical validation.

The fear of missing BM has led some authors to advocate routine LP in infants presenting with an apparent simple FS [26], [40], [49]. In our study, we were not able to assess the utility of routine LP in infants, because 4 of the 7 studies (totalizing 654 children) did not specify the number of such children [10]–[13] (the three other studies included 303 infants [14]–[16]) (Table 1). In Trainor et al.'s study, 13% of the included children were infants [14]. Extrapolation of these data suggests that approximately 85 of the 654 children were infants. Therefore, the total number of infants with an apparent first simple FS in our study could be estimated to be 388 (of whom 223 were enrolled during pre-vaccine era and 165 during post-vaccine era) (Table 1). As only one infant with an apparent first simple FS was found to have CNS infection requiring immediate treatment in our study (Table 1), the NNT to detect one case of such infections would be 388 in these children. This finding challenges the utility of routine LP in infants with an apparent simple FS. However, BM is a rapidly progressive disease and will show itself within a short time, when an LP can be done. A careful clinical observation is necessary in these children during the first few hours after the seizure.

Since introduction of Hib and *S. pneumoniae* vaccines, the incidence of BM has dramatically decreased among young children [50], [51]. As such, the AAP revised its guideline for the neurodiagnostic evaluation of the child with a simple FS in 2011 [52]. The updated AAP guideline no longer supports the routine LP in fully immunized against both Hib and *S. Pneumoniae* infants who present with a simple FS, but recommends an LP as optional in any infants with a simple FS who are missing immunizations or have an indeterminate immunization status [52]. In our study, 223 infants with an apparent first simple FS were enrolled during pre-vaccine era, of whom one was found to have BM (Table 1). The NNT to detect one case of CNS infection requiring immediate treatment could be estimated to be 223 in these children. Accordingly, a careful clinical observation before deciding to perform an LP appears also to be acceptable in infants who are missing immunizations or have an indeterminate immunization status.

Complex FS has been cited as a risk factor for BM [8], [9]. Accordingly, routine LP has been often recommended in the evaluation of such children [53], [54]. However, in our study, the utility of routine LP in children with an apparent first complex FS was low (NNT  = 180). In addition, routine LP following an FS is not devoid of risk. It is sometimes associated with post-LP syndrome which occurs often when LPs have been unnecessary with unpleasant headaches and sometimes vomiting [55], and occasionally may result in fatal cerebellar coning or introduction of organisms from the bloodstream into the CSF [56]. Theses facts challenge the utility of this strategy in such children. Thus routine LP, based solely on complex features of seizure, seems not to be necessary. Short hospital admission for close observation could also be a reasonable strategy in such children.

Our study has some limitations that need to be considered. First, the majority of studies included in our review were retrospective and subject to the biases inherent in that method. Second, in the cases of simple FS and complex FS, BM was ruled out based on clinical grounds alone without CSF examination or clinical examination with follow-up in 43% and 18% of patients, respectively. It is possible that a substantial number of these children who were sent home but who developed meningitis and went to a different hospital may have been missed. However, limiting the analysis to cases in which BM was ruled out by CSF examination or clinical examination with follow-up did not alter the results significantly. Third, the risk of BM in children pre-treated with antibiotics is a diagnostic challenge. Such antimicrobial pre-treatment has been reported to obscure the initial signs and symptoms of BM, and to decrease the rate of CSF culture positivity [57]. As the majority of studies included in this review did not provide data separately on children pre-treated with antibiotics, we calculated the prevalence of BM regardless of absence or presence of antimicrobial pre-treatment. This limits applicability of our results to patients pre-treated with antibiotics. Fourth, while the majority of FS are benign and safely managed at home by the general practitioner [58], more complicated cases of FS with a higher risk of serious infections are referred to hospital. As our study included children seen in the ED or admitted to inpatient ward, our findings may only be applicable to children from similar settings. Finally, the present study was restricted to children from high resource countries, so our findings may not be generalizable to those from low-resource countries.

## Conclusions

The risk of BM presenting solely as an apparent FS is very low, whatever age or features of seizure (simple or complex). Hence, performing routine LP in the absence of any other signs and symptoms suggestive of BM is likely to be of low utility in febrile, young children presenting with a first seizure. Our findings challenge the utility of routine LP in children with an apparent complex FS, and in infants, fully immunized or not against both Hib and *S. pneumoniae*, with an apparent simple FS. A careful clinical observation during the first few hours after the seizure could be an acceptable strategy in such children.

## Supporting Information

Text S1 **PRISMA 2009 Checklist.**
(DOC)

Text S2 **Detailed search strategy for each electronic database.**
(DOC)
