# Supplementary material for: Risk of Bacterial Meningitis in Young Children with a First Seizure in the Context of Fever: A Systematic Review and Meta-Analysis
Source: PLoS One. 2013 Jan 28;8(1):e55270. doi: 10.1371/journal.pone.0055270 (PMC3557257; doi:10.1371/journal.pone.0055270)
Supplement: Text S2 — Detailed search strategy for each electronic database. (DOC) [file pone.0055270.s002.doc]

**Text S2.** **Detailed search strategy for each electronic database**

**MEDLINE via PUBMED**

1. (("fever"[MeSH] OR "fever"[All Fields]) AND ("seizures"[MeSH] OR "seizures"[All Fields] OR "seizure"[All Fields])) AND (("meningitis"[MeSH] OR "meningitis"[All Fields]) OR ("central nervous system infections"[MeSH] OR "central nervous system infections"[All Fields])) AND (("0001/01/01"[PDAT] : "2011/12/31"[PDAT]) AND (English[lang] OR French[lang]) AND ("infant"[MeSH Terms] OR "child"[MeSH Terms] OR "adolescent"[MeSH Terms]))
2. (("fever"[MeSH Terms] OR "fever"[All Fields]) AND ("seizures"[MeSH Terms] OR "seizures"[All Fields] OR "convulsion"[All Fields])) AND (("meningitis"[MeSH Terms] OR "meningitis"[All Fields]) OR ("central nervous system infections"[MeSH Terms] OR ("central"[All Fields] AND "nervous"[All Fields] AND "system"[All Fields] AND "infections"[All Fields]) OR "central nervous system infections"[All Fields])) AND (("0001/01/01"[PDAT] : "2011/12/31"[PDAT]) AND (English[lang] OR French[lang]) AND ("infant"[MeSH Terms] OR "child"[MeSH Terms] OR "adolescent"[MeSH Terms]))
3. ("seizures, febrile"[MeSH Terms] OR ("seizures"[All Fields] AND "febrile"[All Fields]) OR "febrile seizures"[All Fields] OR ("febrile"[All Fields] AND "seizure"[All Fields]) OR "febrile seizure"[All Fields]) AND (("meningitis"[MeSH Terms] OR "meningitis"[All Fields]) OR ("central nervous system infections"[MeSH Terms] OR ("central"[All Fields] AND "nervous"[All Fields] AND "system"[All Fields] AND "infections"[All Fields]) OR "central nervous system infections"[All Fields])) AND (("0001/01/01"[PDAT] : "2011/12/31"[PDAT]) AND (English[lang] OR French[lang]) AND ("infant"[MeSH Terms] OR "child"[MeSH Terms] OR "adolescent"[MeSH Terms]))
4. ("seizures, febrile"[MeSH Terms] OR ("seizures"[All Fields] AND "febrile"[All Fields]) OR "febrile seizures"[All Fields] OR ("febrile"[All Fields] AND "convulsion"[All Fields]) OR "febrile convulsion"[All Fields]) AND (("meningitis"[MeSH Terms] OR "meningitis"[All Fields]) OR ("central nervous system infections"[MeSH Terms] OR ("central"[All Fields] AND "nervous"[All Fields] AND "system"[All Fields] AND "infections"[All Fields]) OR "central nervous system infections"[All Fields])) AND (("0001/01/01"[PDAT] : "2011/12/31"[PDAT]) AND (English[lang] OR French[lang]) AND ("infant"[MeSH Terms] OR "child"[MeSH Terms] OR "adolescent"[MeSH Terms]))
5. (((#1) OR #2) OR #3) OR #4

**INIST (Scientific and Technical Information Institute) via article@inist**

1. fever AND seizure AND child AND (central nervous infections OR meningitis)
2. fever AND convulsion AND child AND (central nervous infections OR meningitis)
3. febrile seizure AND child AND (central nervous infections OR meningitis)
4. febrile convulsion AND child AND (central nervous infections OR meningitis)
5. (((#1) OR #2) OR #3) OR #4

**COCHRANE library**

1. [(fever) and (convulsion) and (child) and (meningitis OR central nervous system infections)](http://onlinelibrary.wiley.com/o/cochrane/searchHistory?mode=runquery&qnum=20)
2. [(fever) and (seizure) and (child) and (meningitis OR central nervous system infections)](http://onlinelibrary.wiley.com/o/cochrane/searchHistory?mode=runquery&qnum=19)
3. [(febrile convulsion) and (child) and (meningitis OR central nervous system infections )](http://onlinelibrary.wiley.com/o/cochrane/searchHistory?mode=runquery&qnum=18)
4. [(febrile seizure) and (child) and (meningitis OR central nervous system infections)](http://onlinelibrary.wiley.com/o/cochrane/searchHistory?mode=runquery&qnum=17)
5. (((#1) OR #2) OR #3) OR #4
